# Histone Modifications in the Cell Cycle of *C. elegans* Embryogenesis: A Comparative Review

**DOI:** 10.3390/epigenomes10010015

**Published:** 2026-02-27

**Authors:** Anati Alyaa Azhar, Hector Mendoza

**Affiliations:** 1 Department of Molecular, Cellular and Developmental Biology, University of Michigan, Ann Arbor, MI 48109, USA; anati@umich.edu; 2 Department of Biology, Loyola University Chicago, Chicago, IL 60660, USA

**Keywords:** epigenetics, histone post-translational modifications, histone methylation, histone phosphorylation, cell cycle regulation

## Abstract

Cell division is a highly regulated process that actively involves dynamic changes to the genetic material within the nucleus. DNA is faithfully replicated in the S-Phase of the cell cycle, being converted from loose, relaxed chromatin into tight, condensed chromosomes to be segregated in mitosis. In addition to scaffolding proteins that shape these mitotic chromosomes, post-translational modifications of histones within nucleosomes modulate chromosome dynamics throughout the cell cycle. In this review, we use a comparative approach to highlight some of the major epigenetic marks affected by the cell cycle during embryogenesis of *Caenorhabditis elegans*: H4K20me1, H3S10ph, H4S1ph, H2AS1ph, and H3T118ph. These five histone post-translational modifications will be specifically highlighted in the context of the mitotic cell cycle, as they are well documented in the *C. elegans* literature.

## 1. Introduction

Epigenetic modifications involve stable alterations to gene expression or phenotype achieved without changing the underlying DNA sequence. These modifications are often subject to mitotic inheritance and, in some cases, to transgenerational (meiotic) inheritance. However, many are transient due to their involvement in regulatory processes and more accurately considered features of chromatin dynamics, as opposed to true epigenetic memory. Modifications, like DNA methylation and modifications of histone proteins, are chemical moieties or ‘tags’ that alter DNA accessibility and chromatin structure, which in turn impact gene expression [1]. These processes have been implicated in the regulation of the cell cycle, a coordinated series of events in which gene expression is primarily impacted by chromatin dynamics through covalent changes to DNA and histones [2,3]. The conventional cell cycle begins with an interphase that consists of three phases: the G1-Phase, in which the cell grows, the S-Phase, in which the genetic material is synthesized or replicated, and the G2-Phase, in which the cell prepares for active nuclear division by synthesizing the necessary organelles and proteins. When checkpoint requirements are met, the cell will enter the M-Phase, in which the nucleus actively divides (karyokinesis) and the cytoplasm is split in two (cytokinesis) to produce genetically identical daughter cells [4,5,6].

Epigenetic modifiers regulate the cell cycle by controlling gene expression, as well as chromatin condensation and chromosome segregation [7,8]. Chromatin is highly dynamic in proliferating cells. In the S-Phase, new histones are incorporated with nascent DNA, creating a requirement for the re-establishment of histone modifications. Chromatin remodelers and the transcriptional machinery dissociate from chromatin during mitosis, yet the cell continues to maintain its transcriptional identity post-mitosis, highlighting the importance of histone modifications and their impact on the progression of the cell cycle. Post-translational modification (PTM) of histones involves the covalent attachment of chemical groups to specific residues that can influence the state of chromatin, which consists of repeating nucleosome units in which DNA segments are wrapped around histone proteins to form a “beads-on-a-string” structure that can fit into the nucleus [9,10]. Histone PTMs include methylation, acetylation, and phosphorylation. Specific patterns of histone modifications make up an “epigenetic code” or language that impacts chromatin dynamics and accessibility, ultimately influencing the transcriptional output of specific regions of the DNA [11].

In *Caenorhabditis elegans*, most active cell division occurs in the first half of embryogenesis, as cells cycle rapidly from the S-Phase to the M-Phase [12]. Apart from two germ cells, the embryonic cells are somatic and divide mitotically. Epigenetic changes vary during this progression, playing a key role in the condensation and segregation of chromosomes, the dissociation of effectors from the chromosomes, and the tight regulation of gene expression [13,14]. Cell cycle regulation in *C. elegans* has been primarily explored from the perspective of cytoskeletal factors like the mitotic spindle, protein scaffolds like condensins that bind to and segregate chromosomes, and essential regulators such as cyclins and cyclin-dependent kinases (CDKs). The field of *C. elegans* research lacks a comprehensive summary of the involvement of epigenetic modifications. In this review, we provide a comparative examination of some of the major histone modifications affecting the cell cycle during *C. elegans* embryogenesis. Based on the available literature, we primarily focus on five epigenetic markers (H4K20me1, H3S10ph, H4S1ph, H2AS1ph, and H3T118ph) and discuss what is known in *C. elegans* compared to other organismal systems. Particular attention will be given to these five specific histone post-translational modifications, analyzing their roles and regulatory mechanisms during the mitotic cell cycle.

## 2. Histone Modifications Can Modulate Chromatin

**Figure 1:**
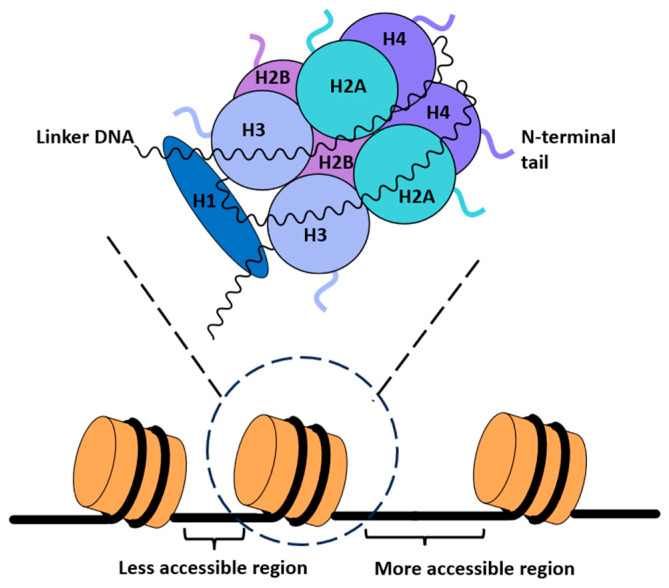
Schematic diagram of nucleosomes and the corresponding core particle. Eight histone proteins (two of each H2A, H2B, H3, and H4) constitute the core particle. Generally, wider spacing between nucleosomes leads to more accessible regions of DNA, while narrower spacing leads to nucleosomes packing closer together, limiting DNA accessibility. However, this relationship is also dependent on many other factors and the impact on gene expression may deviate in species- and tissue-specific contexts.

The DNA present in eukaryotic cells is vast in length but is kept packaged neatly in the nucleus. This higher order packaging of the genetic material is due to nucleosomes, the fundamental repetitive units of chromatin [9]. The nucleosome core particle is an octamer complex of proteins which consists of a centrally positioned H3-H4 tetramer flanked by two H2A-H2B dimers [10]. Each nucleosome has 147 bp of DNA wrapped around a core particle, leading to a ~7-fold compaction of the genetic material present in eukaryotic nuclei [9,15,16]. Nucleosomes are also further stabilized by an H1 ‘linker’ histone located at the entry and exit points of the bound DNA [17]. The length of the DNA associated with the linker histone varies widely among organisms, reaching lengths of up to 100 bp, like in the case of echinoderms [18,19,20], and being as short as 7 bp, like in the case of fission yeast [21]. Structural work has previously associated longer linker DNA with a more open chromatin structure that is less thermodynamically stable, while shorter lengths were found in more compact genomic regions (Figure 1) [22,23]. However, the correlation between linker DNA length and chromatin structure is highly complex, as it must also account for factors like the length of nucleosome repeats, nucleosome positioning, histone modifications, and presence of reader proteins that interact with chromatin [24,25].

The four core histones, H2A, H2B, H3, and H4, range from 11 to 15 kDa and are highly conserved amongst eukaryotes [26]. Each core histone consists of a globular core domain within the nucleosome that shares a similar structural motif with each other: a C’ terminal tail and an unstructured N’ terminal tail that extends away from the nucleosome [9]. The residues in this flexible N’ terminal tail can be modified by covalent post-translational modification, including methylation, acetylation, and phosphorylation. There is a cooperative effort between the ‘writer’ proteins that attach a modifier to histones, ‘reader’ proteins that recognize and bind the modifier to elicit a reaction themselves or through the recruitment of other proteins, and ‘eraser’ proteins that will remove this modifier from histones [27]. This pattern of histone modifications produces a ‘histone code’, or a signature that signifies a specific reaction in the cell regarding overall chromatin state and gene expression [11].

Methylation (me), or the attachment of a methyl group (-CH_3_), occurs predominantly on the basic amino acids lysine (K), arginine (R), and histidine (H). Lysine can be mono (me1), di (me2) or tri (me3) methylated on its ε amine group [28,29,30,31], while arginine can be monomethylated (me1), symmetrically dimethylated (me2s) or asymmetrically dimethylated (me2a) on its guanidine group [32,33]. Histidine, on the other hand, can be monomethylated either at position 1 or 3 of its imidazole ring [34]. The pattern of methylation will determine the functional outcome, allowing for a diverse range of regulatory effects. Methylation is a form of epigenetic regulatory control in many eukaryotes that was once thought to be irreversible due to the stability of this modification compared to others such as phosphorylation [35,36,37]. However, since several lysine demethylases (KDMs) [38,39,40,41,42] and lysine methyltransferases (KMTs) [28,43] have been discovered, it is now recognized that histone methylation can be stably maintained for a long period of time, as in the case of silenced heterochromatin [44,45,46], or can fluctuate during cell differentiation [47,48]. Regulation of histone post-translational modification is directly dependent on the activity of KMTs and KDMs. KMTs are writer proteins that transfer the methyl group from S-adenosyl methionine (SAM) onto lysine residues of histone and non-histone proteins [49]. There are over 30 KMTs fully characterized and while they have impressive target specificity, they are also evolutionarily conserved [50]. KDMs are eraser proteins that remove methyl groups and are distinguished by their highly conserved LSD1 and JmjC domains [38,51,52]. The addition of the methyl group does not modify the charge of the residue and is coined to be the reason that methylation can both promote or repress transcription, depending on the methylation site. Proteins with chromodomains are readers of this methyl modification in which they recognize and bind these methyl groups and carry out the corresponding molecular response.

Phosphorylation (ph) of histones was first described in 1967 [53]. This PTM involves the attachment of a phosphate group (-PO_4_^3−^) and it occurs on histone amino acids that contain hydroxyl groups (-OH), which are serine (S), threonine (T) and tyrosine (Y). Nuclear protein kinases use ATP to phosphorylate these residues and can be counteracted by nuclear protein phosphatases. The negative charge of the phosphate group added to histones reduces the positive charge of the histones, potentially disrupting histone–histone and histone–DNA electrostatic interactions. Presumably, this would lead to the repelling of the negative phosphorylated histone residue to the negatively charged DNA backbone. However, phosphorylation of a specific site is context-dependent in its outcome. For example, the phosphorylation of histone H3 in nucleosomes that localize to inducible promoters allow for a rapid and transient transcriptional response. In another case, histone H3 phosphorylation is associated with chromatin condensation that allows cells to progress into the M-Phase of the cell cycle, as well as with chromatin decondensation post-mitosis. Thus, histone phosphorylation is dynamic in nature and associated with a myriad of processes, including the DNA damage response, transcription regulation, apoptosis, and, most relevant to this review, chromatin compaction [54].

The acetylation (ac) of histones in vivo was first described in 1964 [55]. In acetylation, acetyl groups (-COCH_3_) are added to histone proteins. This modification was originally detected in calf thymus cells in arginine-rich histones [56]. It was noted that the lysine-rich histones were highly acetylated compared to the arginine-rich histone fraction [56]. Acetylation involves the transfer of an acetyl group from acetyl-CoA to the ε amine group of lysine (K) or to the guanidium group of arginine (R), neutralizing their positive charges [55]. This process is reversible and mediated by acetylases [57] and deacetylases [58,59]. Through isotope labeling and mass spectrometry, histone acetylation was confirmed to be highly dynamic, with a high degree of turnover despite the existence of stable acetylation sites [60,61]. When acetylation neutralizes the positive charge of the lysine and arginine side chains, histone–DNA and histone–histone electrostatic interactions are altered. This leads to the loosening of DNA, promoting site-specific transcription. Acetylation marks also serve as recognition sites for bromodomain-containing proteins, an evolutionarily conserved protein–protein interaction module present in many ATP-dependent chromatin remodelers [62,63]; and for YEATS domains, which contain immunoglobulin-like folds that regulate gene expression in yeast and other higher eukaryotes [64]. The diversity of acetylation marks is vast, and, to our knowledge, there are no studies investigating the genome-wide effect of this histone modification in *C. elegans*, making the cell cycle regulation perspective difficult to consider [65].

## 3. The Cell Cycle During *C. elegans* Embryogenesis

*C. elegans* is a microscopic roundworm that exhibits androdioecy, with hermaphrodites determined by two X chromosomes (XX) and males determined by a single X chromosome (XO). This contrasts with species with the XY determination system, in which the Y chromosome triggers male-specific development. *C. elegans* development starts with the embryo encased by a protective eggshell layer before hatching into a larva (the L1 stage). This larva undergoes three additional larval stages (L2, L3 and L4) before reaching sexual maturity, resulting in an adult hermaphrodite or male.

*C. elegans* has an invariant cell lineage in which each animal undergoes the same set of cell divisions at specific time points during development [12]. Most cell divisions occur in the first half of embryogenesis, while, in the second half, the embryo undergoes modifications that affect its external appearance to hatch out of the enclosed egg. Very much like in other metazoans, early embryonic development in *C. elegans* rapidly cycles from the S-Phase to the M-Phase, foregoing gap phases and cell growth to prioritize the increasing cell number [66]. These synchronous early cell divisions can occur due to maternally loaded mRNAs and proteins passed down to the embryo from the hermaphrodite parent. Gap phases are introduced when cell divisions become asynchronous, and their duration is dependent on the cell lineage involved [67]. For instance, the first gap phase introduced in embryonic development is the G2-Phase and is only introduced during the division of intestine primordium cells E2 and E4 once inwards migration of the embryo occurs [66,67]. However, the G2-Phase ends when E4 further divides into E8 cells, showing the tight regulation of these cell cycle phases [67]. Due to the limitations in sensitivity in current assays to establish the different phases of interphase in *C. elegans* embryos, this warrants further research.

After fertilization, a number of cell divisions occur in the newly fertilized egg to produce founder cells, which divide in relatively fixed timeframes [12]. Most of the founder cells are stem cells that are somatic, serving as the progenitors of a particular tissue or organ. The founder cell, P4, generates the germ line, dividing once at the 88-cell stage to produce two primordial germ cells, Z2 and Z3 [12,68,69]. The precursor cells, Z2 and Z3, do not divide in embryogenesis; instead, the somatic gonad precursors, Z1 and Z4, migrate towards them to generate the gonad primordium. Z2 and Z3 then divide during the mid-L1 stage to produce germ cells within the gonad, while Z1 and Z4 undergo cell division to produce the distal tip center (DTC) and somatic gonad cells [70,71].

A DTC caps each of the two gonad arms in the hermaphrodite adult, serving as a stem cell niche to maintain the population of proliferative germ cells adjacent to it [71,72]. When the germline expands during the L3 and L4 stages, the number of proliferative germ cells rapidly increases. The mitotic zone, also known as the proliferative zone, extends from the distal tip to the transition zone (TZ) in which cells enter meiosis. Proximal germ cells farthest from the DTC enter meiosis during the mid-L3 stage [73,74]. An adult hermaphrodite consists of 959 somatic cells and ~2000 germ cells, while an adult male consists of 1031 somatic cells and ~1000 germ cells [75].

## 4. Overview of Histone Modifications During *C. elegans* Embryogenesis

Soon after fertilization, the nucleus of the oocyte undergoes two rounds of meiotic division and then replicates its haploid genome to produce the oocyte-derived pronucleus [76]. The oocyte-derived pronucleus will then fuse with a sperm-derived pronucleus that has also replicated its own haploid genome. Pronuclear fusion occurs as the parental genomes merge at metaphase I, resulting in decondensed chromosomes [77,78]. The single cell embryo transitions from meiotic divisions to mitotic divisions under tight regulation and coordination as meiosis-specific regulators are degraded and replaced by mitosis-specific ones [79,80]. Maternally loaded proteins and RNAs regulate the early cleavages, timing of the first cell divisions, and all events related to the oocyte-to-embryo transition [81]. At the 2-cell stage, chromosomes are positioned randomly, and no epigenetic marks are detected [82]. At the 4-cell stage, zygotic transcription initiates [83]. There is a global reorganization of chromosomes during the 8-cell stage, as epigenetic marks, nuclear domains, and transcriptional activity become evident [82,84].

Histone modifications can also be inherited across generations, establishing an epigenetic ‘memory’ of specific expression patterns in the offspring [85,86,87]. For instance, the acetylation marks H2BK12ac, H3K23ac, H3K27ac, H4K16ac, H4K5ac, H4K8ac and H4K12ac have been previously reported during the 2-cell to 16-cell transition as a result of parental reprogramming pathways [88]. Transient acetylation is also detected during DNA replication as new histones are assembled into chromatin, as observed in *Tetrahymena*, *Drosophila melanogaster* and human cells [89]. Active methylation marks such as H3K4me2 [90] and H3K79me2 [88] are also present on the chromosomes of embryos derived from parental chromosomes [78]. There is also inactive chromatin established as the embryo develops, such as H3K9me2/3 that promotes the formation of heterochromatin [91]. However, these methylation marks prove to be more variable throughout development than the acetylation marks.

The epigenetic landscape of a developing embryo is dynamic, constantly changing in response to genetic and environmental factors. For instance, this can be observed by comparing autosomes of early embryos to those of the L3 stage larva. For both the embryo and larva, actively transcribed chromatin is associated with high levels of H3K4me1, H3K27ac, and H3K36me3, and low levels of H3K4me3 on enhancer regions [92]. On the other hand, silent chromatin is enriched with the Polycomb-mediated silencing mark H3K27me3 and the heterochromatin mark H3K9me3 [92]. Differences can also be detected between the early embryo and the L3 larva. For example, there is a sharp increase in H3K36me3 levels in early embryos, while they are kept at lower levels in the L3 stage [92]. Similarly, the L3 larva has high levels of H3K27me1 on active chromatin domains while they are kept at lower levels in the active chromatin domains of early embryos. There are also differences in the epigenetic profile of the X chromosomes from early embryos compared to the L3 larva stage due to dosage compensation (DC), in which the X chromosomes of hermaphrodites (XX) are both downregulated by ~50% to equalize X-specific expression to that of males (XO).

In the *C. elegans* germline, epigenetic histone modifications play an important role in establishing specific gene expression patterns to ensure proper germline function across generations. For example, the maternal-effect sterile (MES) proteins that are supplied maternally regulate germline development in *C. elegans* through histone PTMs transmitted to embryos [93,94]. MES-4 is a KMT that generates H3K36 dimethylation (H3K36me2) in the autosomes of early embryos and the *C. elegans* germline [95,96]. In *mes-4* mutants, H3K36me2 is abolished and the F1 progeny are sterile, showing the role the transmission of this epigenetic mark plays in functional germline development [95,97,98]. Indeed, *mes-4* mutants have misregulated gene expression in which somatic genes are expressed and germline genes are repressed, contrasting with wild-type [98].

Interestingly, there seems to be an antagonistic relationship between H3K36me3 and H3K27me3 in the germline of *C. elegans*. While H3K36me3 is associated with active transcription, H3K27me2 deposited by the Polycomb Repressive Complex 2 (PRC2) is associated with repressing gene expression. In wild-type *C. elegans*, there are alternating clusters of H3K36me3-bound genes and H3K27me3-bound genes on autosomes in the germline [98] creating active and silent domains, respectively, within the germline. Additionally, the repressive H3K27me3 is also enriched on the silent X chromosomes in the germline. In embryos, loss of H3K36me3 was accompanied by an increase in H3K27me3. However, genes that retained H3K36me3 lacked H3K27me3. This result corroborates findings from other studies [99,100,101], leading to an interesting relationship between opposing histone PTMs that cooperate to regulate gene expression.

As previously mentioned, the epigenetic landscape can also be impacted by environmental factors. Such is the case of worms that are fed a high-fat diet, which have high levels of H3K4me3 near the promoter regions of genes related to lipid metabolism, increasing their transcription [87]. This shows that nutrition can also affect the epigenome by altering chromatin state. Indeed, the impact of environmental stressors on the epigenome, ranging from toxic chemicals to microbial pathogens, represents a trending research field that directly benefits from the use of *C. elegans* as a model organism and warrants its own separate review.

Chromatin is also highly dynamic during the cell cycle. Transcription is generally inhibited during the mitotic phase and this phenomenon is highly conserved across eukaryotes [102]. The epigenome plays a pivotal role as the cells exit mitosis, as it carries the necessary information to restart transcription as the cell continues to grow and develop. Specifically, chromatin is restructured during the S-Phase through the incorporation of newly synthesized histones into nascent DNA, producing a template that is blank from any histone PTMs [103]. Formation of active chromatin is dependent on the reinstatement of the histone modifications transmitted from parent cells to daughter cells. Interestingly, there are histone marks, such as H3K9me2, that are not affected by the cell cycle and are present on both interphase and mitotic chromatin [104]. Contrastingly, other marks like H3S10ph are present in mitosis, specifically during the prophase to telophase transition, before decreasing to undetectable levels as the cell enters the interphase [104,105]. Both H3K9me2 and H3S10ph function as ‘phospho-methyl switches’ that modulate the binding of effector proteins to histone H3, which ultimately impacts the chromatin state [106].

The regulation of histone PTMs during the cell cycle of *C. elegans* is highly complex, since it can originate from diverse inputs like genetic factors, environmental stressors, cell lineage and developmental status. Coupled with the emergence of other overlapping fields, like RNA interference and aging, this further complicates the interpretation of the roles of histone PTMs in mitosis. In this review, we summarize the histone modifications that are affected by the cell cycle in *C. elegans*, with special attention to embryogenesis since somatic proliferation ceases as the animal transitions to adulthood. For this reason, the epigenetic marks we review are H4K20me1, H3S10ph, H4S1ph, H2AS1ph, and H3T118ph. Table 1 highlights the function during the cell cycle of each epigenetic mark in *C. elegans* in contrast to other model organisms. Moreover, we take a comparative approach, highlighting first what is known in other classical organismal systems with the hopes of motivating more research projects centered around epigenetic regulation of the cell cycle.

## 5. H4K20me1 Peaks in Mitosis and Is Enriched on Dosage-Compensated X Chromosomes

Histone H4 methylation is evolutionarily conserved from simple eukaryotes, like yeast [120], to more complex eukaryotic cells, like humans [120,121]. Most of the methylation on histone H4 occurs on lysine 20 (H4K20) [122,123]. H4K20 can be mono (me1) [29], di (me2) [124] or tri(me3) methylated [33,122]. The research field has attempted to establish a downstream effect clearly associated with H4K20me1/2/3, but it has been found that this modification impacts a variety of processes, including transcriptional silencing [125] and activation [108], heterochromatin formation [126], chromatin compaction [120], and mitosis [127]. Since the signal induced in the cell by this modification can be contradictory, the different methylation states of H4K20 are context dependent.

It is reported that H4K20me2 is the most abundant modified form of H4K20 in eukaryotic cells, with ~80% of histones occupied by this mark [128,129]. H4K20me3 lies on the opposite side of this spectrum, being the least abundant form of methylated H4K20 [130,131,132]. Interestingly, both H4K20me2/3 levels do not alter dramatically throughout the cell cycle compared to H4K20me1 [121,133,134]. H4K20me1 also plays an essential role in the establishment of H4K20me2/3, as it has been shown that the abolishment of H4K20me1 leads to the reduction in levels of H4K20me2/3 [135]. In the late G1-Phase, H4K20me1 levels decrease and are lowest during the S-Phase. This may be due to the fact that during the replication of DNA, new unmodified histones are incorporated into the DNA, which dilutes the concentration of the existing histone modifications [136]. After the replication of DNA, H4K20me1 levels begin to increase and reach a peak during mitosis [133,134,135]. Post-mitosis, H4K20me1 is converted into H4K20me2/3, which leads to the production of quiescent cells in the G0/G1 of the cell cycle [133].

Although H4K20 methylation has been detected in several organisms since the 1970s, the protein that writes H4K20me1 was first uncovered in the 2000s. Human PR-Set7 [137] and its *D. melanogaster* ortholog, SET8 [137,138], are KMTs with SET domains (Su(var)3-9, Enhancer-of-zeste and Trithorax). X-ray crystallography experiments confirmed that these KMTs methylate H4K20 and produce H4K20me1 [139,140]. In a similar manner, Suv4-20h1 and Suv4-20h2 were found to di- and tri-methylate H4K20me1 in the mouse model [121], with their counterparts discovered later in humans [129] and *D. melanogaster* [141].

As mentioned earlier, methylation of histones was largely considered a stable process. However, the discovery of a histone demethylase specific to H3K4, LSD1 [38], which is also conserved in both yeast and humans, proved that there is a turnover of histone methylation. In terms of erasers of H4K20 methylation, PHF8 functions as an H4K20me1 demethylase, with a role in the cell cycle in addition to its capability of demethylating H3K9me1/2 and H3K27me2 [142,143,144]. Recently, two human homologous proteins, hHR23A and hHR23B, have been found to demethylate H4K20me1/2/3 in vivo in eukaryotic cells and in vitro through a bulk histone demethylase assay followed by mass spectrometry [145].

In *C. elegans*, SET-1 is one of the smallest SET domain-containing proteins and was found to be an essential gene, as RNAi-mediated gene silencing led to early embryonic death [146]. A decade later, the association between SET-1 and H4K20 methylation was made. Through the RNAi-mediated gene silencing of *set-1*, it was reported that H4K20me1 was abolished in the intestinal nuclei of adult *C. elegans* [110]. This finding was further supported by work with homozygous *set-1* mutant animals, which have no detectable H4K20me [109]. These mutants also developed into sterile adults [109], highlighting the importance of establishing the first methyl moiety on H4K20 to serve as the foundation for H4K20me2/3.

The *C. elegans* ortholog of Suv4-20 is SET-4 and it is the major KMT that converts H4K20me1 into H4K20me2/3. This conclusion was derived from evidence in both homozygous *set-4* mutant embryos and adults, in which H4K20me1 levels increase throughout all chromosomes while H4K20me2/3 levels are reduced [109,110]. This led to an elegant model which summarizes the H4K20 writers in *C. elegans*; SET-1 places the first methyl mark on H4K20, followed by SET-4 that converts it to H4K20me2/3. However, the eraser proteins of H4K20 methylation in this organism are yet to be discovered.

Although the H4K20me1 demethylase in humans have been identified to be PHF8, a homologous counterpart has yet to be found in *C. elegans*. However, a H4K20-specific demethylase that converts H4K20me2/3 to H4K20me1 in this animal has been identified, DPY-21. Originally, DPY-21 was found to be involved in the X chromosome dosage compensation (DC) mechanism of hermaphrodites [147]. This process equalizes gene expression stemming from the sex chromosomes of the differing biological sexes [148,149]. In *C. elegans*, hermaphrodites downregulate both X chromosomes by half to equalize the transcriptional output to that of the single X chromosome present in their male counterparts [150]. This process is conducted primarily by the dosage compensation complex (DCC) [147,151,152,153,154,155], a multimeric complex of proteins that work in tandem to bind and repress the X chromosomes in hermaphrodites.

DPY-21 is one of the DCC subunits [147,156] and has been shown to function as a H4K20-specific demethylase, specifically targeting H4K20me2/3 both in vivo, through immunofluorescent imaging of intestinal nuclei and embryos [110,157], and in vitro, through a bulk histone demethylase assay [157]. DPY-21 removes the methyl groups from H4K20me2/3, leading to the enrichment of H4K20me1 on the X chromosomes and the transcriptional downregulation and compaction of these X chromosomes in the somatic cells of hermaphrodites [110,157,158]. DPY-21’s H4K20me2/3 demethylase activity is limited, as it localizes to the X chromosomes, although weak levels of H4K20me1 on autosomes have been reported [109,110], hinting at the existence of uncharacterized demethylases in *C. elegans*.

As previously mentioned, in eukaryotic cells, H4K20me1 levels decrease during the transition from the G1-Phase to the S-Phase, before increasing again after DNA replication is complete and reaching a peak in mitosis. In post-mitotic cells, H4K20me1 is converted to H4K20me2/3, where it may lay quiescent as the cell prepares to divide again. In *C. elegans* embryonic nuclei, H4K20me1 is low in distribution throughout all chromosomes in interphase but increases when it associates with condensed chromosomes in mitosis, as proven by the high levels of H4K20me1 detected on prometaphase and metaphase chromosomes [109].

H4K20me1 remains evenly distributed throughout all chromosomes as the embryo develops until it reaches the comma stage, in which there are nuclei with H4K20me1 enrichment on the X chromosomes in hermaphrodite embryos due to the DC-silencing mechanism [109]. By the late 3-fold stage, all nuclei in the hermaphrodite embryo display H4K20me1 enrichment on the X chromosomes while H4K20me2/3 is distributed in a relatively uniform manner throughout all chromosomes [109,159]. This enrichment of H4K20me1 on X chromosomes is dependent on the presence of a functional DCC as it is abolished in DC mutants [109]. In male embryos, there is no requirement for DC and immunofluorescent experiments showed uniform distribution of H4K20me1 throughout all chromosomes [109]. Interestingly, H4K20me1 is also present on the inactivated X in female mammalian cells, although the abolishment of this mark does not affect the downregulation of gene expression as it does in *C. elegans* [107]. Instead, it is proposed that H4K20me1 may be associated with the compaction of the inactivated X chromosome.

## 6. H3S10ph Is Associated with Mitotic Chromosome Condensation and Modulates the Binding of Proteins to Chromatin

Early reports on the involvement of histone phosphorylation in the regulation of the eukaryotic cell cycle have been crucial for the development of chromatin remodeling models [160,161,162]. This modification can occur on all four core histones and on the linker histone; however, H3 phosphorylation is of particular importance due to its participation during cell division. These studies provided evidence that H3 phosphorylation was not observed during the interphase periods of the cell cycle but increased during mitosis, specifically in prophase, metaphase, and anaphase, when chromosomes are fully condensed [160]. When cells exit anaphase and chromosomes begin to decondense, H3 dephosphorylation occurs, further supporting that H3 phosphorylation is associated with mitotic chromosome condensation.

Initially, studying phosphorylated histones was troublesome, as the nature of in vitro techniques led to the loss of phosphorylation in isolated chromosomes [163]. This major obstacle was conquered by coupling the isolation of chromosomes to the addition of ATP to ensure maintenance of phosphorylation by endogenous kinases [164]. As a result, H3 phosphorylation was confirmed in mitotic chromosomes, with the major site of phosphorylation being serine 10 (H3S10ph) through tryptic peptide mapping [164]. The discovery of serine 10 as the major phosphorylation site of histone H3 triggered a cascade of studies to characterize this modification, and in terms of mitosis, H3S10ph was found to be correlated to chromosome condensation [111,112,114], while its dephosphorylation was associated with chromosome decondensation [165,166]. These patterns have been observed in both simple [105,111,112] and complex eukaryotic systems [105,160,166]. Exceptions exist, as in the case with *D. melanogaster*, in which strong reduction in H3S10ph only leads to slight decondensation of chromatin [167].

H3S10ph initiates on pericentromeric heterochromatin during the G2-Phase and, as chromosomes condense, the mark spreads throughout all chromosomes [114]. This modification occurs primarily in late-replicating chromatin in which maximal chromosome condensation is obtained during the G2/early prophase transition [168]. In mammals, centromeres are the primary site for the initiation of chromosome condensation [169]. In the G2-Phase, H3S10ph initiates on centromeres before increasing on pericentric heterochromatin, prekinetochores and advancing to the chromosome arms [114]. When premature chromosome condensation is induced in cells, pericentric H3S10 is phosphorylated. However, prekinetochores that are not in the G2-Phase are not fully replicated and lack H3 phosphorylation [169,170]. These results point to the relationship between H3S10 phosphorylation and the condensing of mitotic chromosomes, as well as the replication of genetic material for the cell to undergo division. When cells arrested in the early S-Phase are induced to undergo mitosis and forego the G2-Phase, less than 1% of cells exhibit proper mitotic chromosome condensation [168]. The few cells that enter mitosis exhibit H3S10ph, highlighting that the accumulation of this modification in G2-Phase is essential for mitotic entry [168]. The dephosphorylation of H3S10 begins in late anaphase and early telophase and is completed just before chromosomal decondensation post-mitosis [114,168,171]. Thus, H3S10ph is required for mitotic chromosome condensation, mitotic entry, and its dephosphorylation is associated with the decondensation of chromosomes post-mitosis.

Following the discovery of H3S10 phosphorylation and its association with mitosis in eukaryotic cells, H3S10ph was then established to be a mitotic mark in *C. elegans*. In embryos, as well as in L1 larvae, H3S10ph is observed on mitotic chromosomes from prometaphase to telophase [113]. Since then, H3S10ph has been used as a mitotic mark in several *C. elegans* studies [105,172,173]. Interestingly, H3S10ph is also present in the mitotic region of the hermaphrodite gonads, located between the distal tip cells (DTC) and the transition zone to meiosis. In the nuclei of this mitotic region, H3S10ph is present on metaphase and telophase chromosomes, parallel to what is observed in embryos, before becoming undetectable in meiosis [105].

In addition to its role for proper mitotic chromosome condensation, H3S10ph is tightly linked to H3K9me2. H3K9me2 is adjacent to H3S10ph, and these two residues have been hypothesized to function as a ‘phospho-methyl switch’ to regulate the binding of histone H3 to effector proteins [174,175,176]. H3K9me2 is required for the nuclear periphery localization of heterochromatin [48,104]. CEC-4 is a chromodomain-containing protein in *C. elegans* that anchors H3K9 methylation-bearing chromatin to the nuclear periphery in interphase [177,178,179]. When H3K9 is di-methylated, its reader protein CEC-4 tethers it at the nuclear lamina [106]. However, the mutation H3S10E, which mimics a constitutively phosphorylated state, displaces H3K9me2-marked chromatin from the nuclear periphery [104]. Contrastingly, the mutation H3S10A which is non-phosphorylatable had no effect on the localization of H3K9me2-marked chromatin to the nuclear lamina [104]. Indeed, it is reported that the binding of recombinant, purified CEC-4 to methylated H3K9 peptides decreases significantly when the adjacent H3S10 is phosphorylated [106]. From these in vitro biochemical assays, the phosphorylation of H3S10 can prevent reader proteins, including CEC-4 from reading and binding to H3K9me2. However, in vivo assays are required to establish that this is translatable within a model organism as well.

Before cells enter mitosis, H3K9me2-localized chromatin detaches from the nuclear periphery and remains in the nucleoplasm. Once the cell enters prophase, the nuclear lamina is disassembled and H3S10ph increases. H3K9me2 remains present on both interphase and mitotic chromosomes while H3S10ph is localized only to mitotic chromosomes. Thus, it is proposed that when H3S10ph increases in mitosis, it inhibits the binding of effector proteins to H3K9me2, preventing H3K9me2 readers/tethers to anchor H3K9me2-marked chromatin to the nuclear periphery [180,181]. As the cell enters telophase, H3K9me2-marked chromatin is reestablished on the nuclear lamina. H3K9me2S10ph-marked chromatin remained in the nucleoplasm, suggesting that dephosphorylation of serine 10 occurs before reassociation of the H3K9me2-marked chromatin with the nuclear lamina [104].

In addition to the H3K9me2/H3S10ph ‘switch’, H3S10ph also prevents the binding of heterochromatin protein 1 (HP1) to heterochromatin [182]. During interphase, HP1 binds to H3K9me3-marked heterochromatin domains before it decreases in prophase until it becomes undetectable in prometaphase as observed through immunofluorescent microscopy of mouse embryonic fibroblasts [181]. This research group found that H3S10ph and HP1 localization to chromosomes occur at alternating times. When H3S10 phosphorylation is inhibited, HP1 remains enriched on pericentric heterochromatin during mitosis. In an in vitro assay, HP1 binds to H3K9meS10ph less efficiently than it does to H3K9me3 peptides [177,181]. It is proposed that the negative charge of phosphorylated serine 10 repels the negatively charged glutamate 52 residue in HP1. Thus, H3S10ph is able to regulate the binding of proteins to chromatin as in the case of CEC-4 to methylated H3K9 and HP1 to H3K9me3-marked heterochromatin in vitro, providing a foundation for further in vivo studies.

H3S10 phosphorylation is associated with mitosis, and this modification modulates effector proteins that bind to chromatin. Additionally, this mitotic marker also plays a role in the maintenance of a stable genome, as observed in studies with *Saccharomyces cerevisiae* that was then extended to human cells and *C. elegans*. R-loops are an indicator of genomic instability [183,184]. In yeast mutants, R-loop accumulations exhibit increased H3S10ph [183]. When this research group expanded their study to include *C. elegans*, R-loop-accumulating mutant worms also had elevated levels of H3S10ph and, interestingly, H3K9me2 in both mitotic and meiotic regions of the hermaphrodite germline. Since H3S10ph is associated with chromosome condensation, the research group also reported that the nuclei in these mutants were highly condensed compared to wild-type. Thus, H3S10ph is associated with chromosome compaction in not only eukaryotic cell lines but also in the *C. elegans* mitotic and meiotic germline.

The H3S10ph ‘writer’ also plays an important role in cell cycle regulation. When kinase activity for H3S10ph is outcompeted with the transfection of H3S10 peptides into cells, entry to mitosis is prevented. Cells that managed to enter mitosis despite the transfection exhibited H3S10ph, proving the importance of the phosphorylation state of H3S10 [168]. Preliminary in vitro assays identified a cAMP-dependent kinase as the enzyme responsible for H3S10ph [185], paving the way for subsequent in vivo studies that further characterized the pathways involved [186,187]. In 2002, the kinase IpI1p and the phosphatase Glc7p were identified in *S. cerevisiae* as the writer and eraser proteins for H3S10 phosphorylation through genetic assays and in vitro biochemical assays [105]. This same research group expanded their studies into *C. elegans*, discovering that AIR-2, an Aurora B kinase involved in cell division, serves as the kinase that phosphorylates H3S10. This was observed when the RNAi-mediated silencing of *air-2* abolished H3S10ph in embryos, leading to embryonic arrest at the 1-cell stage [105]. This same silencing also affected mitotic nuclei in hermaphrodite gonads, leading to aberrant mitotic divisions with decondensed chromatin [105]. These results show that genetically, AIR-2 is required for the phosphorylation of H3S10. However, in vitro biochemical assays are necessary to determine if AIR-2 is able to directly phosphorylate H3S10.

The discovery of AIR-2 as a kinase involved in H3S10 phosphorylation in *C. elegans* led to the identification of its H3 phosphatase counterpart. In budding yeast, Glc7p is a type 1 protein phosphatase (PP1) that removes the phosphate group from H3S10 [105]. In *C. elegans*, two isoforms encode for PP1, Ceglc-7α and Ceglc-7β. In worms with a loss of H3S10ph, disruption of either isoform through RNAi led to a rescue of H3S10ph [105]. Thus, Ceglc-7α and Ceglc-7β are both able to dephosphorylate H3S10. However, there are differences amongst these two isoforms. The silencing of Ceglc-7α led to 100% embryonic lethality in which embryos are arrested at late stages, exhibiting misshapen nuclei consisting of abnormal amounts of DNA due to defective chromosome segregation. On the other hand, the silencing of Ceglc-7β leads to ~50% embryonic lethality, with all surviving embryos arrested at the 50–100 cell stage. When the silencing of both Ceglc-7α and Ceglc-7β was coupled, there was 100% embryonic lethality and arrest at the 50–100 cell stage [105]. Taken together, these results suggest that both isoforms may be involved in additional pathways.

Additionally, various studies have expanded on PP1, identifying several of its protein interactors, such as the human factor C1 (HCF) implicated in viral transcription [188]. The *C. elegans hcf-1* deletion mutant has small brood sizes and embryonic lethality. Interestingly, the *hcf-1* mutants are defective in cell division due to mitotic spindle defects and misalignment [172]. In these *hcf-1* mutant embryos, H3S10ph is significantly reduced. This loss of H3S10ph is reproducible in mammalian cells with depleted HCF-1 [172]. However, when Ceglc-7α and Ceglc-7β were silenced through RNAi in *hcf-1* mutant *C. elegans* embryos, two contrasting phenotypes are observed: embryos with elevated H3S10ph as well as embryos that lacked H3S10ph. The research group proposes that HCF-1 may play a role in the maintenance of H3S10ph in a pathway different than that of the Ceglc-7α/Ceglc-7β phosphatase. However, this could also be due to knockdown inefficiency through RNAi-mediated silencing. Thus, more research is needed to expand the relationship between HCF-1 and H3S10ph.

To summarize, H3S10ph is associated with mitotic chromosome condensation and its dephosphorylation with chromosome decondensation. The accumulation of this histone PTM also helps the cell cycle progress from the G2-Phase to the M-Phase. Additionally, it can regulate the binding of proteins including CEC-4 and HP1 to its neighboring residue, methylated H3K9, at least in vitro. Studies in *C. elegans* have thus far utilized H3S10ph widely as a marker of mitosis and there is research that shows it is correlated to the condensing of chromosomes in both the mitotic and meiotic regions of the hermaphrodite germline. The multifaceted roles of H3S10 phosphorylation, as revealed by current research, underscore its biological significance and highlight the need for further in-depth studies to fully elucidate its functions.

## 7. H4S1ph Is Biphasic Throughout the Cell Cycle and Peaks in Mitosis

H4S1 phosphorylation was initially detected in newly synthesized histones, in which the phosphorylation and acetylation of residues on H4 were determined to be transient in dividing cells [189]. The first five amino acids of the N’ terminal tail of H4 are conserved amongst most eukaryotic organisms, with a few exceptions like in *Tetrahymena* [115]. This N’ terminus area also exhibits frequent modifications, in which arginine 3 (H4R3) is methylated during transcriptional activation [190] and lysine 5 (H4K5) is acetylated on newly synthesized histones [89,189]. Moreover, phosphorylation of H4 occurs on serine 1 (H4S1) according to mass spectrometry data [115].

Phosphorylated H4S1 was found to be present in asynchronous eukaryotic cells. When studied further, there was a drastic increase in phosphorylated H4S1/H2AS1 in cells arrested in mitosis, denoting the link of this mark with mitosis [115]. The specific timing of H4S1 phosphorylation was established through the immunofluorescent imaging of synchronous HeLa cells at specific time points [115]. H4S1ph increases at the G1/S-Phase checkpoint and in early S-Phase but decreased to basal levels during the mid S-Phase to the early G2-Phase. Interestingly, H4S1 is once again phosphorylated nearing mitosis, where it reaches a peak. It is maintained at high levels until the end of telophase and the early G1-Phase [115]. Thus, H4S1ph exhibits a biphasic pattern of distribution in which it increases in G1-Phase/early S-Phase, falls to basal levels in mid S-Phase/early G2-Phase, before reaching a peak in mitosis. This biphasic distribution of H4S1ph is compelling; however, it has only been shown in HeLa cells. Since the distribution of histone PTMs may vary between cell lines as well as organisms, the pattern of H4S1ph reported is limited and warrants further research to establish if this is indeed true for other systems.

Interestingly, in non-mitotic cells, there is a low level of H4S1ph detected due to phosphorylation of H4S1 in newly synthesized and deposited histones [115,189]. Although histone modifications are more commonly nuclear in nature, there are also modifications that occur in the cytoplasm, including acetylation and phosphorylation. Presence of H4S1ph in non-mitotic cells may be due to the phosphorylation of the residue occurring early in histone biosynthesis while the nascent polypeptide chains are still attached to polysomes in the cytoplasm [115,189,191]. Through the labeling of purified erythrocyte histones, it was found that in newly synthesized histones, H4S1 is modified by phosphorylation or acetylation before later removal [189]. This finding has been limited to in vitro biochemical assays and would benefit from more in vivo studies to establish if H4S1 is indeed phosphorylated in the cytoplasm during synthesis.

As previously mentioned, H3S10ph is considered a hallmark of mitosis. In mitotic chromosomes of *C. elegans*, H3S10ph was detected at high levels in early embryos undergoing [115]. In *C. elegans* embryos, phosphorylated H4S1 is present on the condensed chromosomes of prophase and metaphase, colocalizing with H3S10ph. This mitotic-specific pattern of H4S1 phosphorylation is reproducible in other organisms such as *Physarum polycephalum* [115]. There are exceptions to this cell cycle-specific pattern of H4S1ph. For instance, in *D. melanogaster*, the first residue of H4 is a threonine (H4T1) instead of a serine [115]. Both serine and threonine are polar amino acids with neutral charges, but threonine has an additional methyl group that makes it bulkier. H4T1ph was detected in chromosomes in metaphase as well as in the S-Phase [115]. This low level of H4T1 phosphorylation in the S-Phase may be due to the phosphorylation of newly synthesized histones that are necessary for the stable incorporation of histones into nascent chromatin [89,189]. This deduction requires further experimentation in order to provide more insights on the role of this phosphorylation mark in cell cycle regulation.

In yeast, H4S1 is conserved but there is no mitotic phosphorylation of this mark [115]. Instead, it is present in basal levels throughout the cell cycle of this eukaryote [192]. Since its discovery in mitosis, H4S1ph has been associated with DNA damage repair, the regulation of chromatin acetylation [193], transcription [194] and yeast sporulation [194]. Casein Kinase II (CKII) has been shown to phosphorylate H4S1 in vitro [195]. Additional work is needed to prove CKII activity in vivo. Interestingly, research has revealed that there is a meiotic role associated with H4S1ph in which it is conserved during gametogenesis in several organisms including yeast, *D. melanogaster*, and mice [196]. In terms of meiosis, H4S1ph promotes chromosomal compaction and transcriptional downregulation [194,196]. Since the only research done in *C. elegans* concerning H4S1ph is its localization in mitotic nuclei of young embryos, exploring this modification during germline development and maintenance could shed light on potential ‘writers’, ‘readers’, and ‘erasers’.

## 8. H2AS1ph Increases in Mitosis and Is Implicated in Transcription Regulation In Vitro

Amongst the four core histones, H2A has the largest sequence divergence, creating multiple H2A family proteins and a myriad of variants amongst organisms [197]. Like H4, the first five residues of H2A are sequentially conserved across various eukaryotes and maintained throughout evolution [115,198]. The number of phosphorylation sites on H2A have been detected and multiple functions are associated with these modifications. For example, phosphorylation of H2A at serine 129 occurs in response to DNA damage to facilitate DNA repair in *S. cerevisiae* [199,200]. In *S. pombe*, H2A serine 121 is phosphorylated for the tethering of the protein Shugoshin to centromeres, preventing chromosomal instability [201].

H2A serine 1 has been found to be the residue most commonly phosphorylated based of mass spectrometric data in human cells [202]. The distribution of H2AS1 phosphorylation throughout the cell cycle has been characterized in several systems, including *C. elegans*, *D. melanogaster,* and HeLa cells [115]. H2AS1ph exhibits the same biphasic pattern as described earlier in H4S1ph in which levels in the G1-Phase/early S-Phase transition, fall to basal levels in mid S-Phase/early G2-Phase and peak in mitosis [115]. Since the first five residues of the amino terminal in both histone H4 and histone H2A are conserved, this may explain the same levels of distribution displayed by both. H2AS1ph is high in mitosis but there are low levels of it detected in S-Phase, which could also be due to H2AS1ph on newly synthesized histones. However, this has only been reported for H4S1ph and not H2AS1ph, which warrants more research [189,203,204]. Interestingly, mouse embryonic fibroblasts do not exhibit a strong biphasic pattern of H2AS1 as previously reported in HeLa cells [115]; however, H2AS1ph levels appear to be highest in mitotic cells, which corroborates previous findings [204].

In addition to the distribution of H2AS1 phosphorylation throughout the cell cycle, research has also uncovered some of its protein regulators. HMGN1 is a nuclear protein that binds nucleosomes and reduces the compaction of chromatin fiber [205], which alters transcription, replication, and repair [205,206,207]. Using mouse embryonic fibroblasts, as well as through in vitro biochemical assays, the loss of HMGN1 increases H2AS1 phosphorylation, while HGMN1 binding to nucleosomes inhibits it [203,204]. Mitogen- and stress-induced kinase (MSK1) phosphorylates H2A, and this negatively regulates transcription in vitro [203]. However, since this finding is restricted to in vitro reconstitution assays, more research needs to be conducted to demonstrate that MSK1 phosphorylates H2AS and if this modification results in reduced gene expression. Protein Phosphatase 2Cγ (PP2Cγ) has been shown to bind H2AS1ph [208]. However, this assay is limited to the physical interaction during the co-immunoprecipitation of PP2Cγ and H2AS1ph. More experiments, such as H2AS1 peptide phosphorylation assays and mutational screens, need to be conducted in order to determine if PP2Cγ directly phosphorylates H2AS1.

Histone H2A has many variants, which are non-allelic isoforms with different primary sequences and temporal expression, such as H2A.X and H2A.Z [209]. This adds another layer of complexity in studying H2AS1ph. Moreover, this epigenetic mark has not been explored extensively in *C. elegans* and in the context of the regulation of the cell cycle. The characterization of H2AS1ph regulators, like HGMN1 and MSK1, represent excellent starting points for *C. elegans* work. Interestingly, in *Xenopus*, H2 Serine 1 is phosphorylated concomitant with germinal vesicle breakdown, which is when meiosis resumes in oocytes but the modification is independent of DNA replication and cell cycle transitions [210]. This finding may also serve as the foundation of studies regarding maintenance of the *C. elegans* germline by H2AS1ph.

## 9. H3 T118ph Ensures Chromosome Alignment and Proper Shaping of Mitotic Chromosomes

The phosphorylation of histone H3 on threonine 118 (H3T118ph) was initially detected through mass spectrometry of mammalian core histones [211,212]. This residue is located within the globular domain of H3, positioned at the interface of the histone and bound DNA. H3T118 is conserved amongst many eukaryotic species [118] and in the last decade, has been implicated to have ties to the cell cycle [119,211].

Initially, H3T118 was studied in the context of SIN (Swi-INdependent) mutants in yeast. SWI/SNF complexes reorganize chromatin structure, enabling binding of other proteins to nucleosomal DNA to influence transcription [213,214,215]. SIN mutants allow transcription while foregoing the SWI/SNF complex. One of the SIN mutants, *SIN2*, was later identified as a mutation of histone H3 in yeast [216,217], converting threonine 118 to isoleucine 118 [116,117]. Threonine 118 is positioned on the surface of the H3-H4 tetramer and is oriented close to the bound DNA and can form hydrogen bonds. When H3T118 is mutated to H3I118, protein-DNA interactions are disrupted, leading to nucleosome dissociation [218,219]. This shows that H3T118 plays an important role within the nucleosome structure. Not only is the H3T118 residue itself important, but its phosphorylation is also an essential component. In yeast, the phosphomimetic mutation H3E118 and the non-phosphorylatable mutation H3A118 are lethal [118]. H3 T118ph enhances nucleosome mobility, which allows proper repositioning of nucleosomes in response to physiological temperature fluctuations [220]. However, these results are limited to in vitro biochemical assays, limiting their translational potential in a more biological context.

Many studies on H3T118 phosphorylation are related to nucleosome stability and mobility within chromatin. At present, only one research group has studied H3T118ph in relations to the mitotic cell cycle, with a large focus on cell lines [7,119]. During the cell cycle, there is an increase in H3T118ph levels in mitotic eukaryotic cells [119]. Immunofluorescence experiments demonstrate that H3T118ph levels increase from prophase to anaphase but are largely reduced during interphase [119]. In mitosis, H3T118ph forms discrete foci on chromatin in prophase, pro-metaphase and on the spindle midbody during anaphase [119]. In both *D. melanogaster* and *C. elegans*, H3T118ph localizes to chromatin and centrosomes in mitosis [119]. Interestingly, in *C. elegans* embryos during pro-metaphase, H3T118ph is also detectable on the periphery of the holocentric chromosomes where centromeres are located [119].

When H3T118ph is mutated, there are mitotic defects. For instance, in human cells transfected with mutant H3T118ph, there is improper chromosome alignment in metaphase, lagging chromosomes that fail to move to the cell poles in anaphase, and delayed cytokinesis [119]. Additionally, these H3T118ph mutations cause the loss of sister chromatid arm cohesion and produce shorter chromosomes [119]. Since the shaping of mitotic chromosomes involves chromosome scaffolding proteins, the research group focused on how altering H3T118ph affects any of these scaffolding proteins. Indeed, when H3T118ph is mutated, there is a loss of Condensin I occupancy on chromatin followed by longer chromatin loops observed in SEM. Interestingly, the study also reports that the localizations of H3T118ph and Condensin I are distinct although they appear on chromosomes at similar periods [119]. Additionally, in vivo biochemical assays show that Condensin I binds to nucleosomes regardless of the phosphorylation state of H3T118. When the kinase responsible for H3T118 phosphorylation was overexpressed, it was reported that Condensin I occupancy on mitotic chromosomes was significantly reduced. From these results, the research group hypothesizes that H3T118ph regulates Condensin I binding to chromatin, thereby influencing chromosome packing. However, these findings are limited and specific to HeLa cells, probing the question if this is reproducible in other cell lines and/or in other species.

In terms of physiological importance, the lethality of H3T118ph yeast mutants is reproducible in *D. melanogaster*. All H3 gene copies were deleted from *D. melanogaster* and replaced with transgenes containing mutated H3T118ph [119]. Animals bearing the wild-type H3 transgenes survived until adulthood while animals expressing the mutant H3T118ph transgenes all died as embryos after depletion of the maternal contribution of histones. This shows that H3T118ph is also crucial for the development of both yeast and *D. melanogaster.* Since H3T118ph is conserved in various other eukaryotic species including *C. elegans*, these results compel the question of whether this histone PTM plays a crucial role in the embryonic development of other organisms.

The same research group also attempted to identify the main kinase responsible for the phosphorylation of H3T118 through both in vitro and in vivo assays [119]. In a phosphorylation screen, Aurora A was the only cell cycle kinase that exhibited high phosphorylation activity of the H3T118 peptide [119]. When aurora A kinase was inhibited or knocked down, H3T118ph was diminished from mitotic chromosomes [119]. However, it was also reported that the inhibition of Aurora B kinase ultimately prevents Aurora A kinase from localizing to chromatin in order to phosphorylate H3T118 [119]. Thus, the research group successfully showed that Aurora A kinase is able to directly phosphorylate the H3T118 residue; however, the role of Aurora B kinase remains unclear [119]. Taken together, these results show the complexity of phosphoregulation, as kinases often have multiple substrates.

As stated above, research on H3T188ph has largely been focused on yeast and mammalian cell lines. In *C. elegans*, H3T118ph is observed on mitotic chromosomes of young embryos, specifically from prophase to anaphase before it diminishes in interphase [119]. Since studies have shown that H3T118 phosphorylation is also involved in chromosome alignment as well as the proper shaping of mitotic chromosomes, it would be interesting to explore the relationship of H3T118ph and Condensin I in *C. elegans*. Additionally, since aurora A is a kinase that phosphorylates H3T118, its *C. elegans* orthologue, AIR-1, should stimulate further experimentation.

## 10. Concluding Remarks

**Figure 2:**
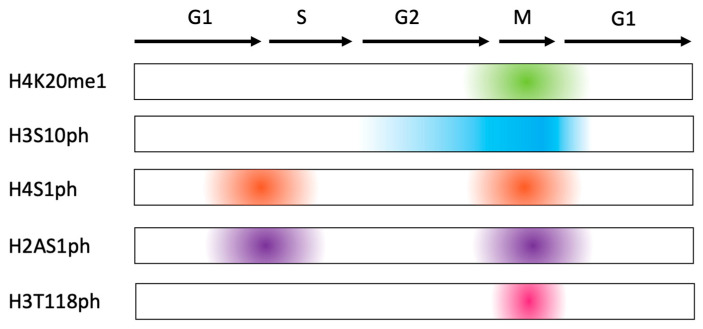
Histone modifications throughout the cell cycle of *C. elegans* embryogenesis. Arrows indicate cell cycle progression. Colors represent presence of the histone mark in the corresponding stage of the cell cycle. These modifications are predominantly found in mitosis based on the available bibliography.

Post-translational modification of histones play an important role in the regulation of the cell cycle of *C. elegans* embryos. However, as illustrated in Figure 2 and based on the available literature, most of what is known about these epigenetic modifiers focuses on mitosis. Although H4K20me2/3 are stably distributed throughout the cell cycle, H4K20me1 levels are low in interphase and peak in mitosis. In *C. elegans*, hermaphrodites (denoted with ‘XX’) downregulate both X chromosomes to equalize to the single X chromosome present in their male counterparts (denoted with ‘XO’) [150]. The dosage-compensated X chromosomes in hermaphrodite somatic cells of *C. elegans* are enriched with the repressive mark H4K20me1, which plays a role in chromosome compaction and gene downregulation [110,157,158]. Contrastingly, for other organisms such as female mammals with two X chromosomes, H4K20me1 decorates the inactivated X chromosome but does not play a role in gene downregulation [107]. Instead, it is proposed that H4K20me1 is associated with the compaction of the silenced X chromosome in female mammalian cells, similar to that of *C. elegans* hermaphrodite cells [107].

In eukaryotic cells, H3S10ph is associated with a variety of functions in which it is essential for mitotic chromosome condensation, mitotic entry, maintenance of a stable genome, dissociation of effector proteins from chromatin and, along with H3K9me2, acts as a ‘phospho-methyl switch’. H3S10ph is a hallmark of mitosis and thus has been used as a mitotic mark in many eukaryotic systems including human cells, *Xenopus*, and *C. elegans*. In eukaryotic cells, the role of phosphorylation of H3S10 in mitosis has been linked to chromosome condensation, while its dephosphorylation leads to chromosome decondensation following the end of mitosis. This epigenetic mark has also been shown to be essential for mitotic entry, as cells are arrested at the G2/M checkpoint when H3S10ph is abolished in eukaryotic cell systems. However, there are exceptions to the role of H3S10ph in organisms such as *D. melanogaster*, in which the removal of H3S10ph does not lead to significant decompaction in chromosomes. This brings to question whether H3S10ph is also involved in mitotic entry and chromosome condensation in *C. elegans*. However, the relationship of this mark to condensed mitotic chromosomes in *C. elegans* has not fully been explored as assays with H3S10ph have been limited to the localization of this mark to pericentromeric chromosomes, similar to eukaryotic cells, before it spreads throughout the chromosomes as the cell cycle progresses.

In HeLa cells and mouse fibroblasts, H4S1ph and H2AS1ph exhibit a biphasic pattern in the cell cycle [115]. H4S1ph/H2AS1ph increases in G1-Phase/early S-Phase, falls to basal levels in mid S-Phase/early G2-Phase, before reaching a peak in mitosis and being dephosphorylated again [115]. However, there are still differences, as mouse fibroblasts do not exhibit a strong biphasic pattern as observed in HeLa cells [204]. Additionally, although H4S1ph is conserved across organisms, *D. melanogaster* has H4T1ph instead, and in fly embryos, H4T1ph is detected during both mitosis and the S-Phase [115]. Research regarding H4S1ph/H2AS1ph is limited to the localization of the modifications to mitotic chromosomes in *C. elegans* embryos. Additionally, the biphasic nature of these two marks has yet to be confirmed in *C. elegans*. More research is needed in additional eukaryotic model systems to have a more complete picture of how these marks are involved in the regulation of the cell cycle.

In several cell lines including HeLa cells, cancer cells and human breast and lung cells, H3T118ph increases in mitosis and localizes to centrosomes and the spindle midbody. H3T118ph work has been done primarily in eukaryotic cells, linking it to chromosome alignment and the proper shaping of mitotic chromosomes. It has also been shown to be crucial in development, as its disruption is lethal in yeast and *D. melanogaster*. In *C. elegans*, H3T118ph increases in mitosis and localizes to centromeres on the holocentric chromosomes of *C. elegans* [119]. However, this modification’s role throughout the *C. elegans* cell cycle in terms of chromosome alignment and condensing of chromosomes has not been further explored. Since H3T118ph plays a role in the proper development of yeast [118] and flies [119], it would be interesting to determine if this mitotic-restricted histone PTM is also essential for the survival of *C. elegans* embryos.

As summarized in this review, H4K20me1, H3S10ph, H4S1ph, H2AS1ph, and H3T118ph are histone PTMs that are affected by the cell cycle in distribution. These modifications each exhibit different patterns of distribution but increase most during the M-Phase of the cell cycle. Moreover, not all histone PTMs are influenced by the cell cycle, as seen from the perspectives of H4K20me2/3 and H3K9me2. Thus, the role of histone PTMs in the cell cycle is highly complex. *C. elegans* represents a promising model system to elucidate and examine the chromatin landscape throughout the eukaryotic cell cycle.

## Figures and Tables

**Table 1:** Mitotic histone marks as reported in multiple species with associated functions and distribution in the *C. elegans* model system.

Histone Modification	Species	Associated Functions	Distribution in *C. elegans*
H4K20me1	Mammalian	Present on inactivated X chromosomes in female somatic cells [107] and transcription regulation [108]	Decreased in G1-Phase to mid S-Phase [109]; increased on Pro-Metaphase and Metaphase [109]; enriched on dosage-compensated X chromosomes of hermaphrodites [109,110]
H3S10ph	*Tetrahymena*	Mitotic chromosome condensation [111,112]	Prophase to Anaphase [105,113]; Metaphase and Telophase in mitotic region of hermaphrodite gonads [105]
	HeLa cells	Mitosis [114]	
	Yeast	Mitosis and Meiosis [105]	
H4S1ph	Mammalian	Mitosis [115]	Increased in Prophase and Metaphase [115]
	*D. melanogaster*	Mitosis and Interphase [115]	
H2AS1ph	Mammalian	Mitosis [115]	Increased in Prophase and Metaphase [115]
	*D. melanogaster*	Mitosis and Interphase [115]	
H3 T118ph	Yeast	Transcription regulation [116,117] and development [118]	Decreased in interphase [119]; increased in Prophase, Pro-Metaphase, Anaphase [119]
	HeLa cells	Mitotic chromosome segregation [119]	
	*D. melanogaster*	Development [119] and mitosis [119]	

## Data Availability

No new data were created or analyzed in this study. Data sharing is not applicable to this article.
